# Impact of periprocedural myocardial injury after transcatheter aortic valve implantation on long-term mortality: a meta-analysis of Kaplan-Meier derived individual patient data

**DOI:** 10.3389/fcvm.2023.1228305

**Published:** 2023-11-10

**Authors:** Mauricio Felippi de Sá Marchi, Pedro Calomeni, Mateus de Miranda Gauza, Gabriel Kanhouche, Lis Victória Ravani, Caio Vinicius Fernandes Rodrigues, Flávio Tarasoutchi, Fábio Sandoli de Brito, Josep Rodés-Cabau, Nicolas M. Van Mieghem, Alexandre Abizaid, Henrique Barbosa Ribeiro

**Affiliations:** ^1^ Departamento de Cardiologia Intervencionista e Hemodinamica, Instituto do Coracao (InCor), Hospital das Clinicas HCFMUSP, Faculdade de Medicina, Universidade de Sao Paulo, Sao Paulo, Brasil; ^2^ Department of Interventional Cardiology, Erasmus Medical Center, Rotterdam, Netherlands; ^3^ Universidade Regional de Joinville, Joinville, Brasil; ^4^ Unidade Clinica de Valvopatias, Instituto do Coracao (InCor), Hospital das Clinicas HCFMUSP, Faculdade de Medicina, Universidade de Sao Paulo, Sao Paulo, Brasil; ^5^ Department of Cardiovascular Medicine, Quebec Heart and Lung Institute, Laval University, Quebec City, QC, Canada; ^6^ Department of Cardiovascular Medicine, Institut Clinic Cardiovascular, Hospital Clinic de Barcelona, Barcelona, Spain

**Keywords:** aortic stenosis, transcatheter aortic valve implantation, transcatheter aortic valve replacement, periprocedural myocardial injury, biomarkers, valvular heart disease, structural heart disease

## Abstract

**Background:**

Periprocedural myocardial injury (PPMI) frequently occurs after transcatheter aortic valve implantation (TAVI), although its impact on long-term mortality is uncertain.

**Methods:**

We performed a pooled analysis of Kaplan-Meier-derived individual patient data to compare survival in patients with and without PPMI after TAVI. Flexible parametric models with B-splines and landmark analyses were used to determine PPMI prognostic value. Subgroup analyses for VARC-2, troponin, and creatine kinase-MB (CK-MB)-defined PPMI were also performed.

**Results:**

Eighteen observational studies comprising 10,094 subjects were included. PPMI was associated with lower overall survival (OS) after two years (HR = 1.46, 95% CI 1.30–1.65, *p* < 0.01). This was also observed when restricting the analysis to overall VARC-2-defined PPMI (HR = 1.23, 95% CI 1.07–1.40, *p* < 0.01). For VARC-2 PPMI criteria and VARC-2 troponin-only, higher mortality was restricted to the first 2 months after TAVI (HR = 1.64, 95% CI 1.31–2.07, *p* < 0.01; and HR = 1.32, 95% CI 1.05–1.67, *p* = 0.02, respectively), while for VARC-2 defined CK-MB-only the increase in mortality was confined to the first 30 days (HR = 7.44, 95% CI 4.76–11.66, *p* < 0.01).

**Conclusion:**

PPMI following TAVI was associated with lower overall survival compared with patients without PPMI. PPMI prognostic impact is restricted to the initial months after the procedure. The analyses were consistent for VARC-2 criteria and for both biomarkers, yet CK-MB was a stronger prognostic marker of mortality than troponin.

## Introduction

Transcatheter aortic valve implantation (TAVI) is a well-established treatment for the management of severe aortic stenosis across the entire spectrum of surgical risk (1, 2).

Periprocedural myocardial injury (PPMI) is a common procedural complication, often evaluated by the release of cardiac biomarkers, as ischemic symptoms in the periprocedural setting are often misleading and confounding in nature (3).

The Valve Academic Research Consortium 2 (VARC-2) defines PPMI as a periprocedural cardiac biomarker, by either troponin or creatinine kinase-MB (CK-MB) elevation, not meeting the criteria for myocardial infarction, with threshold cutoff points of 15× the upper limit of normal (ULN) for troponin and 5× the ULN for CK-MB (4). As troponin assays progressively become more sensitive, the significance of PPMI should be carefully assessed. Notably, in the recently published VARC-3, the proposed cutoff points for both troponin (70× the ULN) and CK-MB (10× the ULN) were significantly higher (5). Therefore, questions remain regarding the prognostic impact of PPMI and its long-term impact.

Previously published meta-analyses on the prognostic relevance of PPMI after TAVI provided limited information on long-term mortality, as they aggregated data on heterogeneous fixed time points, which may result in overlooked patterns and outcome variability (6–8). Furthermore, their results should be viewed with caution, as central tenets of survival analysis are either not recognized or cannot be checked in traditional meta-analyses (9–11). Hence, to address these limitations, this study aimed to determine the prognostic significance of PPMI after TAVI using a pooled analysis of Kaplan-Meier (KM) estimated individual patient data (IPD) of VARC-2 studies or studies with comparable definitions, since, to the best of our knowledge, there is only one published study based on VARC-3 (12).

## Methods

### Eligibility criteria, databases and search strategy

This study followed the Preferred Reporting Items for Systematic Reviews and Meta-analyses (PRISMA) reporting guideline (13). Studies were included if the following criteria were fulfilled: (1) Population comprised patients who underwent TAVI; (2) Reported cardiac-specific biomarker elevation within 72 h; (3) Standardized thresholds cut-points for PPMI based on VARC-2 (peak troponin ≥15 × ULN or CK-MB ≥5 × ULN) or similarly comparable definitions (4); (4) KM curves of all-cause mortality; (5) Fully published status; and (6) Written in English.

PubMed, EMBASE, and Cochrane Center databases were systematically searched for articles that met our inclusion criteria and were published by February 25, 2023. Additionally, we reviewed the references of the included articles and previous reviews to identify relevant texts. We utilized the following search strategy: (“Myocardial injury” OR “PPMI” OR “Troponin” or “Troponin I [TnI]” or “Troponin T [TnT]” or “High-Sensitivity Troponin I [hsTnI]” or “High-Sensitivity Troponin T [hsTnT]” OR “CKMB” OR “CK-MB” OR “Creatine kinase” OR “Creatine phosphokinase” OR “CPK” OR “phosphocreatine kinase”) AND (“Transcatheter aortic valve implantation” OR “TAVI” OR “Transapical aortic valve replacement” OR “TAVR”).

The following steps were taken for study selection: (1) identification of titles of records through database search; (2) removal of duplicates; (3) screening and selection of abstracts; (4) assessment for eligibility through full-text papers; and (5) final inclusion in the study. Two independent reviewers (P.C. and M.M.G.) selected the studies. When there was disagreement, a third reviewer (M.F.S.M.) decided to include or exclude the study. Ethical approval was not applicable to this study as it consisted of a systematic review and meta-analysis.

VARC-2 standardized thresholds were utilized in 13 out of 18 studies (3, 12, 14–24). The other 5 studies utilized the following criteria: CK-MB and/or TnT rise > 5 ULN (25); hsTnT ≥ 166 pg/ml (26); CK-MB > 7 ng/ml (27); TnT increase > 3 ULN (28); and hsTnT rise ≥ 18.3 ULN (29).

### Assessment of the risk of bias

The risk of bias was evaluated using the Risk of Bias In Non-randomized Studies of Interventions (ROBINS-I) (30). The studies and their characteristics were classified as having low, moderate, serious or critical risk of bias. Two independent reviewers (P.C. and M.M.G.) assessed risk of bias.

### Statistical analysis

Time-to-event outcomes are not amenable to the standard statistical procedures. For meta-analyses, pooling the treatment effect over several studies must either use estimates of median survival and event rates assessed from survival estimates at given time points, or fall back on direct estimates of the hazard ratio. These approaches are unsatisfactory since they fail to consider the central principles of survival analysis, such as censoring and the proportional hazards assumption (11, 31). As a consequence, the “curve approach” has emerged as the current gold standard for meta-analysis of time-to-event data (32). This approach reconstructed individual patient data (IPD) based on published KM graphs from the included studies. In this meta-analysis, we used the R package “IPDfromKM’’ version 0.1.10 (33).

Raw data coordinates (time and survival probability) for each treatment arm were extracted from published KM survival curves using dedicated software. Subsequently, data coordinates were processed based on the raw data coordinates from the first stage in conjunction with the numbers at risk at given time points when available, and IPD was reconstructed using the IPDfromKM software.

Quality assessment of KM derived IPD data was performed graphically by comparing the derived KM curves with the original curves. The reconstructed IPD was then merged to create the study dataset.

We visually assessed the outcomes of interest in both arms using KM estimates, next, hazard ratios (HRs) with 95% CIs for the difference between both arms were calculated using a Cox frailty model. The inclusion of a γ frailty term was used to account for heterogeneity between studies, with studies modelled as a random effect using random intercepts. The proportional hazards assumption of the Cox model was checked using the Grambsch-Therneau test and diagnostic plots based on Schoenfeld residuals (34).

To deal with proportional hazards assumption violations and assess how the prognostic value of post-TAVI PPMI changed over time, we performed two complementary techniques. First, we fitted a flexible parametric survival model with B-splines. The baseline hazard rate was modeled on a spline with four degrees of freedom. Interactions between the treatment arm and time were added by using a second spline function. We also added a γ frailty term to account for heterogeneity between studies. This technique allowed us to estimate time-varying hazard ratios for our analyses of interest. Finally, we performed landmark analyses to further discriminate short- and long-term PPMI prognostic values.

Subgroup analyses were performed for VARC-2-defined PPMI, and for the VARC-2 cutoff of both troponin and CK-MB-defined PPMI, to further assess the differences in mortality according to the different biomarkers and to investigate the effect of the VARC-2 cutoffs on mortality over the long-term follow-up.

All analyses were performed using R Statistical Software (version 4.2.2, Foundation for Statistical Computing, Vienna, Austria).

## Results

Our systematic search identified 847 potential articles and one additional record was identified through other sources. There were 31 articles selected for further eligibility assessment after screening the abstracts. All articles were retrieved and reviewed at the full-text level for possible inclusion. The search strategy is shown in Supplementary Figure S1. After further revisions and exclusions, eighteen observational studies that met all eligibility criteria were included in our meta-analysis (3, 12, 14–29).

A total of 10,094 patients were included, the main characteristics of the studies and their patients are presented in Table 1. The mean age was 81 years and 50% of the patients were men. Coronary artery disease and diabetes mellitus prevalence were 53% and 28%, respectively. Transfemoral (TF) approach was used in approximately 90% of all procedures. Valve type was similar between the PPMI and non-PPMI groups, with 50% of both groups using self-expandable valves (SEV) and 47% using balloon expandable valves (BEV). The median follow-up period of our reconstructed time-to-event population was 12 months (IQR: 6–16 months). The incidence of overall VARC-2 defined PPMI was 53%. The incidence of troponin-defined PPMI (61%) was almost sevenfold higher than CK-MB-defined PPMI (9%).

**Table 1:** Overview of the included studies with relevant characteristics.

Study	Population, n^a^	Troponin-defined PPMI/CK-MB-defined PPMI	Age, years^b^, PPMI/No PPMI	Male, % PPMI/No PPMI	HTN, % PPMI/No PPMI	DM, % PPMI/No PPMI	CAD, % PPMI/No PPMI	Previous MI, % PPMI/No PPMI	AF, % PPMI/No PPMI	SEV, % PPMI/No PPMI	BEV, % PPMI/No PPMI	Mean LVEF, % PPMI/No PPMI	TF Approach, % PPMI/No PPMI	Follow-up, years^b^	Definition of PPMI
Akodad et al. (14)	805^c^	366/NR	NR	NR	NR	NR	NR	NR	NR	NR	NR	NR	NI	1	hscTnT > 15 ULN
Barbash et al. (27)	103	NR/37	84/85	43/40	91/97	27/34	45/57	10/16	37/48	NR	NR	57/51	100/100	1	CK-MB > 7 ng/ml
Chorianopoulos et al. (26)	151	78/NR	NR	NR	NR	NR	NR	NR	NR	NR	NR	NR	100/100	1	hscTnT ≥ 166 pg/ml
Dagan et al. (15)	370	242/NR	83/82	50/62	76/69	23/42	39/41	NR	30/43	74/67	25/32	NR	94/99	5	cTnI > 15 ULN
De Marzo et al. (3)	596	471/NR	83/83	40/54	NR	25/37	41/56	16/23	33/37	32/22	57/77	55/55	92/96	1	cTnI ≥ 15 ULN
Filomena et al. (16)	106^d^	40/NR	82/81	10/29	35/61	13/21	NR	7/12	NR	30/16	36/24	51/46	NR	2	hsTnT > 15 ULN
Kohler et al. (17)	216	77/NR	NR	NR	NR	NR	NR	NR	NR	NR	NR	NR	NR	2	hsTnT > 15 ULN
Koifman et al. (18)	473	363/37	83/83	45/63	93/95	11/17	72/75	NR	NR	29/24	71/76	NR	100/100	1	cTn > 15 ULN or CK-MB > 5 ULN
Koskinas et al. (19)	577	338/NR	82/82	47/43	85/83	26/28	67/58	15/14	28/30	47/63	52/36	54/52	67/98	2	cTnT > 15 ULN
Nara et al. (20)	126	82/NR	85/83	14/38	79/65	22/36	7/15	8/15	26/20	22/11	78/89	62/54	96/95	1	cTnI ≥ 15 ULN
Rahhab et al. (21)	1,054	785/NR	85/83	47/60	NR	NR	NR	NR	NR	79/NR	76/NR	60/55	NR	30d	hsTnT > 15 ULN
Real et al. (12)	1,394	817/NR	NR	NR	NR	NR	NR	NR	NR	NR	NR	NR	NR	1	Troponin > 15 ULN
Ribeiro et al. (22)	1,131	NR/108	81/80	45/51	NR	NR	56/53	NR	29/26	44/41	55/58	60/56	NR	2	CK-MB > 5 ULN
Schindler et al. (29)	1,331	322/NR	81/80	52/54	77/77	23/26	55/46	9/9	18/19	45/49	38/35	53/53	89/97	2	hs-cTnT ≥ 18.3 ULN
Sharma et al. (28)	510	376/NR	81/81	54/60	94/94	42/48	NR	NR	NR	NR	NR	NR	47/85	3	cTnT ≥ 3 ULN
Sinning et al. (23)	276	144/25^e^	81/80	50/59	NR	NR	69/59	15/14	36/39	89/79	10/20	53/44	92/97	1	cTnT ≥ 15 ULN or CK-MB ≥ 5 ULN
Stundl et al. (24)	756^d^	390/55	81/80	46/59	NR	26/30	61/60	7/13	39/46	58/64	14/25	57/49	96/97	5	hsTnT > 15 ULN or CK-MB > 5 ULN
Yong et al. (25)	119^d^	NR/20^e^	83/80	50/37	45/51	10/29	30/18	10/20	25/34	100/100	0/0	NR	100/100	1	cTnT > 5 ULN or CK-MB > 5 ULN

n, number; d, days.

^a^ Data extracted from Kaplan-Meier curves at time 0, unless otherwise indicated. NR, not reported; ULN, upper limit of normal; PPMI, periprocedural myocardial injury.

^b^ Mean or median years of follow-up, unless otherwise stated; HTN, hypertension; DM, diabetes mellitus; CAD, coronary artery disease; MI, myocardial infarction; AF, atrial fibrillation; SEV, self-expanding valves; BEV, balloon-expandable valves; LVEF, left ventricular ejection fraction; TF, transfemoral; hscTnT, high-sensitive cardiac troponin T; CK-MB, creatine kinase-myocardial band; cTnI, cardiac troponin I; cTnT, cardiac troponin T.

^c^ Data extracted from 1-month mark in Kaplan-Meier curves

^d^ Data extracted from Table 1, due to the Kaplan-Meier curve not presenting data at time 0.

^e^ Study reported number of CK-MB-defined PPMI, but did not report Kaplan-Meier curve for this subgroup.

### Quality assessment

The ROBINS-I tool identified ten studies as having a low risk of bias and eight studies as having a moderate risk of bias (Supplementary Figure S2). Quality assessment of KM-derived IPD data by visual comparison of the derived KM curves with the original curves did not show any relevant differences. This analysis, therefore, indicates the results derived from this meta-analysis are grounded on well-conducted observational studies and, therefore, should be regarded as more reliable compared with a scenario in which the aggregated studies were mostly at high-risk for bias.

### Pooled analysis for overall survival

**Figure 1:**
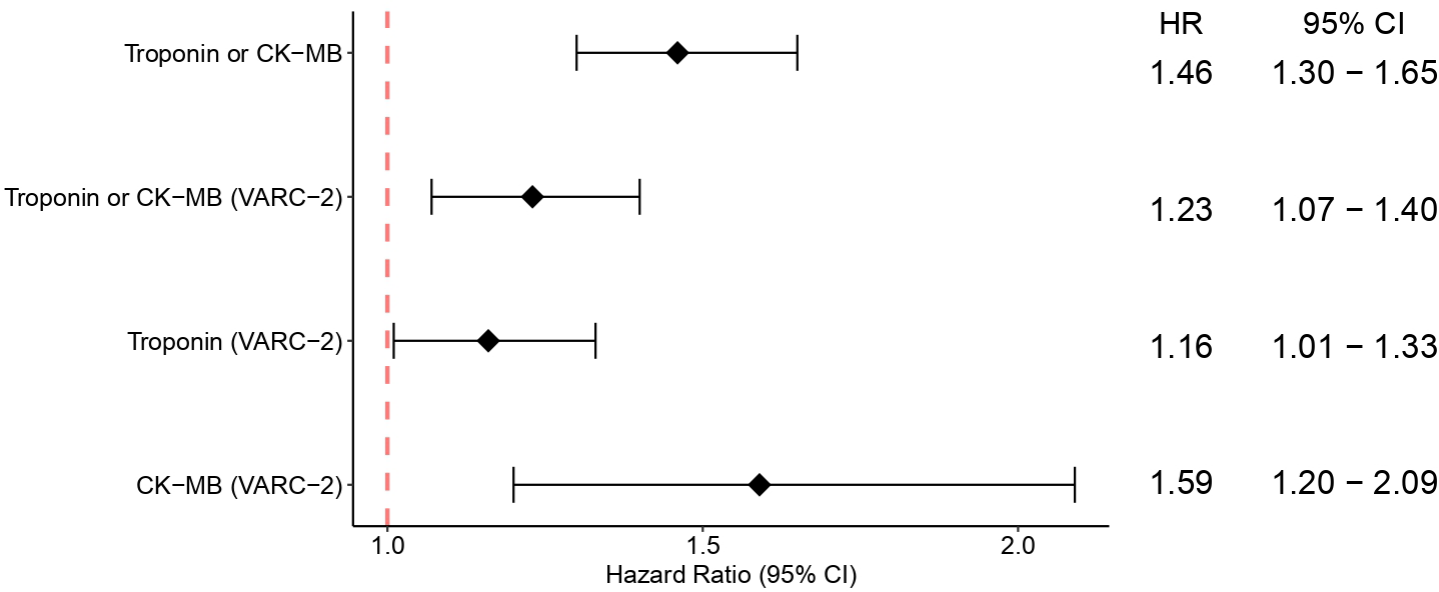
Pooled analysis showing the overall survival following TAVI for PPMI and no PPMI, and for subgroups of VARC-2, VARC-2 troponin-defined PPMI and VARC-2 CK-MB-defined PPMI. CI, confidence interval; HR, hazard ratio; PPMI, periprocedural myocardial injury; TAVI, transcatheter aortic valve implantation.

The pooled analysis for overall survival (OS) comparing patients who had PPMI with those who did not have PPMI after two years revealed that PPMI after TAVI associated with lower cumulative OS (HR = 1.46, 95% CI 1.30–1.65, *p* < 0.01). Likewise, when only VARC-2 criteria were considered, patients with PPMI also had a lower OS than those without PPMI (HR = 1.23, 95% CI 1.07–1.40, *p* < 0.01). To investigate the effects of different myocardial injury biomarkers on PPMI, we performed the same analysis with VARC-2 troponin-defined PPMI and VARC-2 CK-MB-defined PPMI. In both cases, OS at two years was lower in the PPMI group (HR = 1.16, 95% CI 1.01–1.33, *p* = 0.04, and HR = 1.59, 95% CI 1.20–2.09, *p* < 0.01, respectively), yet the association was much stronger with CK-MB than with troponin (Figure 1).

### Landmark analyses and time-varying hazard ratio analyses

**Figure 2:**
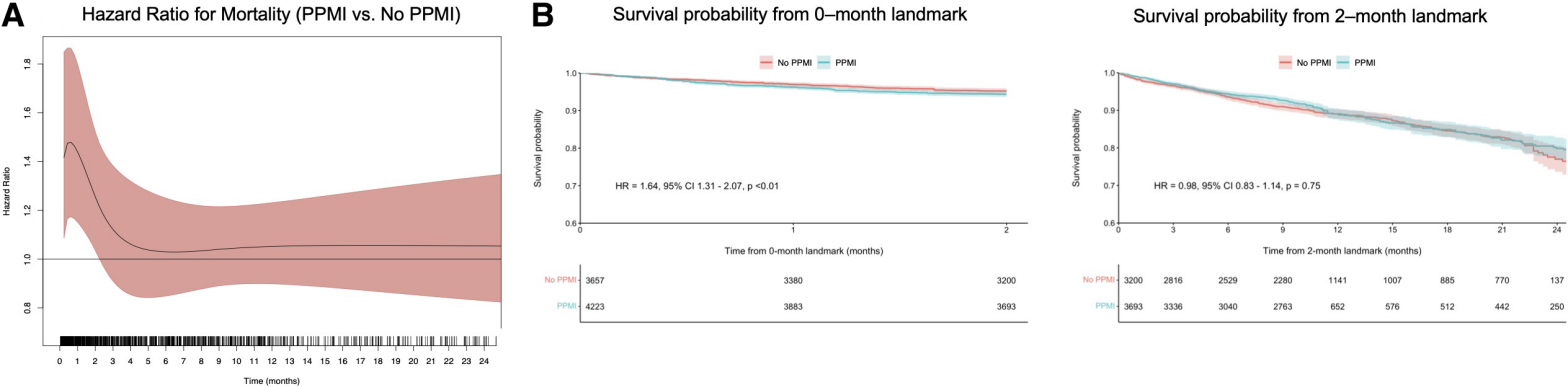
Time-varying HRs with 95% CI for all-cause mortality for patients with VARC-2-defined PPMI compared with no PPMI, at every given time during follow-up; these are derived from flexible parametric survival models with B-splines (**A**) and landmark analysis of overall survival in VARC-2-defined PPMI compared with no PPMI as defined by either troponin or CK-MB, designating 2 months of follow-up as the landmark time (**B**). Abbreviations as in Figure 1.

**Figure 3:**
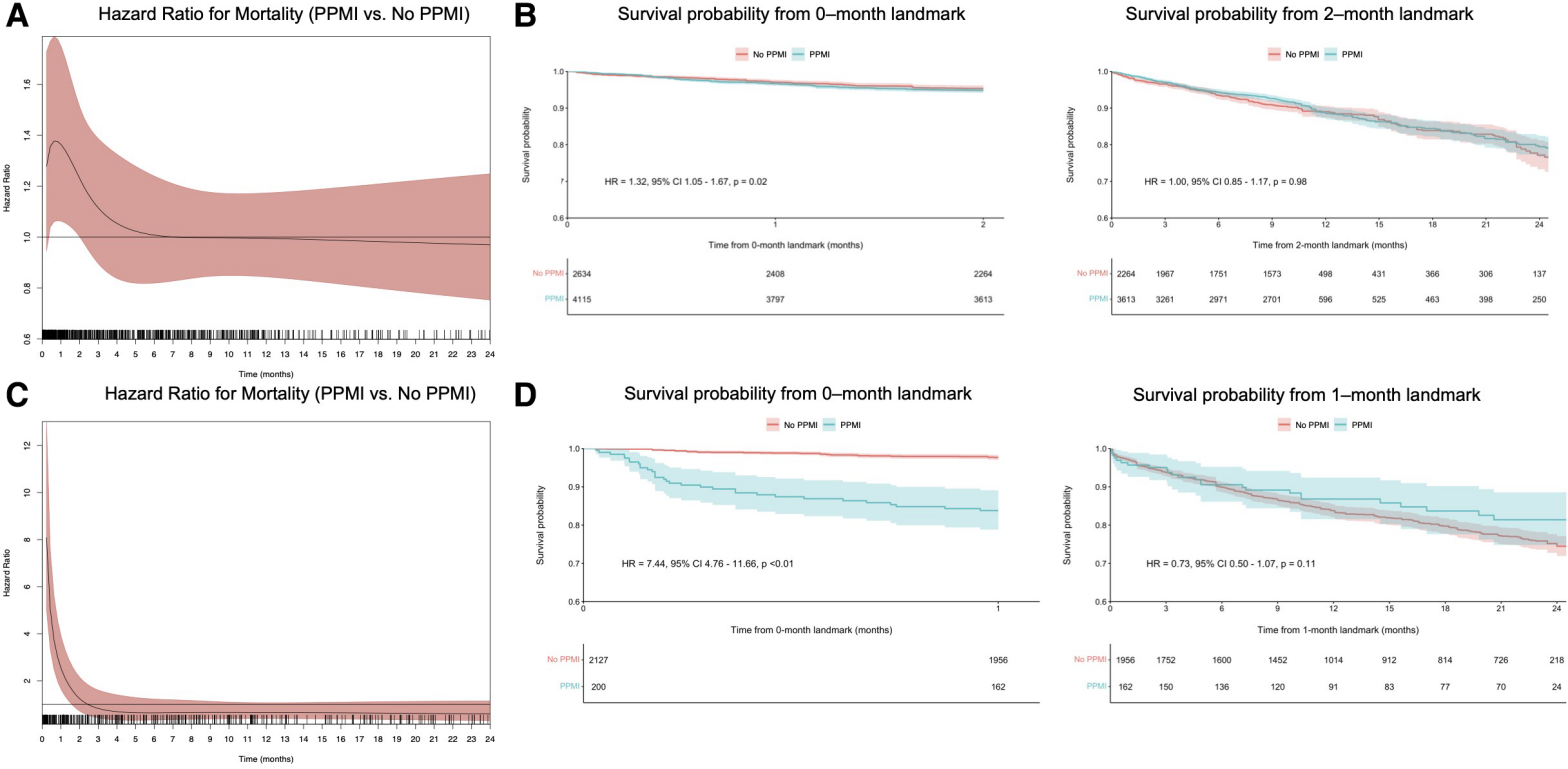
Time-varying HRs with 95% CI for all-cause mortality for patients with VARC-2 troponin-defined PPMI compared with no PPMI, at every given time during follow-up; these are derived from flexible parametric survival models with B-splines (**A**), landmark analysis of overall survival in VARC-2 troponin-defined PPMI compared with no PPMI, designating 2 months of follow-up as the landmark time (**B**), time-varying HRs with 95% CI for all-cause mortality for patients with VARC-2 CK-MB-defined PPMI compared with no PPMI, at every given time during follow-up (**C**) and landmark analysis of overall survival in VARC-2 CK-MB-defined PPMI compared with no PPMI, designating 1 month of follow-up as the landmark time (**D**). Abbreviations as in Figure 1.

Using flexible parametric models with B-splines, we estimated time-varying HRs for VARC-2-defined PPMI, as well as for VARC-2-troponin and CK-MB-defined PPMI. This revealed that VARC-2 and troponin-PPMI were associated with lower OS in the initial two months (Figures 2A, 3A), whereas CK-MB-PPMI was associated with lower OS in the first month only (Figure 3C**)**.

Furthermore, landmark analysis was performed using cutoff values determined by time-varying HRs. In the first two months (Figure 2B), VARC-2 PPMI was significantly associated with lower OS (HR = 1.64, 95% CI 1.31–2.07, *p* < 0.01). However, this was no longer observed after 2 months in the landmark analysis (HR = 0.98, 95% CI 0.83–1.14, *p* = 0.75). The same trend was observed in the subgroup of troponin-only defined PPMI (Figure 3B), and CK-MB only defined PPMI (Figure 3D). In the first two months, troponin-defined PPMI was significantly associated with lower OS (HR = 1.32 95% CI 1.05–1.67, *p* = 0.02), but no longer after the 2 month-landmark (HR = 1.00, 95% CI 0.85–1.17, *p* = 0.98). Finally, in the first month, CK-MB-defined PPMI was strongly associated with lower OS (HR = 7.44, 95% CI 4.76–11.66, *p* < 0.01), but this association was not statistically significant after 1 month (HR = 0.73, 95% CI 0.50–1.07, *p* = 0.11).

## Discussion

In this systematic review and meta-analysis of 18 observational studies, the prognostic value of PPMI after TAVI for longer-term mortality was investigated. The main findings were as follows: (1) PPMI after TAVI was significantly associated with lower overall survival at 2 years; (2) the analysis remained consistent when performed in separate subgroups for VARC-2-defined PPMI and for both VARC-2 troponin- and CKMB-defined PPMI; (3) most deaths occurred within the first 2 months after the procedure; and (4) CK-MB defined VARC-2 criteria for PPMI was a much stronger mortality prognostic marker compared to troponin.

### Incidence and predictors of PPMI

TAVI is a minimally invasive procedure that does not involve aortic cross clamping and cardioplegia, which are established factors for increased cardiac biomarkers release after valvular surgical procedures (35). Nevertheless, prior studies have demonstrated some degree of elevation of both CK-MB and troponin after the procedure in up to two-thirds of TAVI patients (22). Interestingly, PPMI incidence differs according to the cardiac injury biomarker analyzed and the cutoff point used; although troponin elevation > 15 ULN is of common occurrence during the first 72 h post-TAVI, only 10% of patients experience CK-MB elevation > 5 times the ULN (18). Our pooled analysis corroborates these findings, as the incidence of troponin PPMI was 61% vs. 9% for CK-MB defined PPMI, according to VARC-2 criteria of >5 times the ULN for CK-MB and >15 times the ULN for troponin. This difference in the incidence can be partially explained by the fact that CK-MB elevation requires a greater myocardial injury compared with troponin. For instance, as previously shown CK-MB VARC-2 cutoff threshold of >5 ULN displayed a better correlation with troponin levels of >75 ULN, which is much higher than the established VARC-2 recommendation of >15 times (4, 18). Therefore, the optimal PPMI cutoff point remains a matter of debate and, with the advent of ultra- and of high-sensitivity biomarkers assay kits even lower thresholds of myocardial injury can be measured, potentially overestimating the incidence of PPMI, ultimately jeopardizing its clinical relevance (36). Nonetheless, due to the new VARC-3 definition (≥70 times the ULN of troponin), we hypothesize that PPMI incidence will decrease in future studies while its prognostic significance will rise. This was recently demonstrated in a study by Real et al., in which PPMI incidence using troponin was 14% based on the VARC-3 criteria vs. 59% with VARC-2 (12).

PPMI is likely the result of several factors, such as transient hypotension during ventricular rapid pacing, distal microembolization of calcium particles during balloon dilatation and valve manipulation, mechanical compression of left ventricular outflow, subclinical ventricular trauma due to the wire, coronary artery disease that increases oxygen supply-demand mismatch, and coronary artery occlusion (3, 14, 29, 37). Several procedural predictors of post-TAVI PPMI are also known, such as early experience, first generation valves and transapical (TA) approach (5, 22). TA access is not only associated with PPMI, but is also a known factor for apical myocardial necrosis (38, 39). These findings further corroborate the use of alternatives accesses other than the TA, whenever TF access is not feasible (40). Regarding valve types and PPMI, self-expanding valves (SEV) were previously associated with a two-fold higher incidence of PPMI as compared with balloon-expandable valves (BEV), even after adjusting for several possible confounders (3, 21). This might be explained by various reasons, such as balloon pre-dilatation and after SEV deployment, which can lead small calcium particles to embolize to the coronary arteries, myocardial stunning triggered by more events of rapid pacing performed during the additional balloon dilations in comparison with BEV and perivalvular myocardial compression (41, 42).

### Clinical impact of PPMI

Previously published meta-analyses found that PPMI was associated with an increased risk of early and late overall mortality (6–8). Our meta-analysis supports these findings and contributes to the existing literature by aggregating a significantly larger number of patients than previous analyses, indicating that most of the prognostic value of troponin-defined PPMI occurred within the first two months after TAVI, and even earlier for CK-MB-defined PPMI (first month). Furthermore, CK-MB was a better prognostic marker of short and 2-year mortality in comparison with troponin. Two important messages from these results are that first CK-MB using the VARC-2 definition of 5× the ULN is a valuable prognostic tool for mortality. Second, the VARC-2 definition for troponin-defined PPMI of 15× the ULN could overestimate the prevalence of PPMI and hinder its prognostic capacity. VARC-3 definition of 70× the ULN of troponin perhaps is a more suitable value and this is also corroborated by the recent publication of Real et al. which showed no association between VARC-2-troponin defined PPMI with the 1-year mortality (12). Yet, when the analysis was repeated using the VARC-3 cutoff, a statistically significant association was found (12). Importantly, whether the new cutoff is optimal remains uncertain and further studies with larger number of patients, using various assays, and with longer-term follow-up are required to confirm such findings. However, no study to date has specifically indicated potential measures which could improve PPMI patient's prognosis. Still, postprocedural cardiac biomarkers levels evaluation should be used to enhance early months risk assessment, indicating those in need for intensive postprocedural care such as a closer follow-up, possibly within a dedicated TAVI Heart Team, with intensive treatment of risk factors (8, 29).

## Limitations

Our study has limitations that should be considered when interpreting the results. First, only observational studies were included, which are prone to confounders and other biases. Second, this is a meta-analysis of KM derived IPD. We do not have access to patient-level data, which would allow us to minimize the risk of confounding effects through statistical techniques and to assess specific patient or procedural characteristics that could affect the clinical outcomes. Third, there is significant heterogeneity between studies, due to the different biomarker assay kits used and the evolution in the TAVI bioprostheses, technique and operator experience over time. Finally, some studies did not exclusively perform TF TAVI, which warrants special attention when considering PPMI rates and outcomes, as non-TF approaches are associated with higher PPMI rates and worse outcomes. Unfortunately, TA patients subgroup analysis was not possible in our study as TA approach effect on PPMI was not systematically described in the revised literature.

## Conclusions

In this meta-analysis of 18 observational studies with 10,094 patients included, PPMI after TAVI was associated with lower OS as compared with no PPMI. This was consistent for both troponin-defined PPMI and CK-MB-defined PPMI. Time-varying hazard ratios and landmark analyses revealed that most of the prognostic power of the biomarkers, with respect to mortality, ensued in the first months after the procedure. Altogether, these results suggest that PPMI is an important prognostic marker in the acute phase following the procedure. Finally, given the more sensitive troponin assays currently in use, VARC-3 recommendations seem more suitable to determine clinically relevant PPMI than VARC-2, pending larger studies to confirm such findings.

## Data Availability

The original contributions presented in the study are included in the article/**Supplementary Materials**, further inquiries can be directed to the corresponding author.

## Supplementary material

SUPPLEMENTARY FIGURE S1

PRISMA flow diagram of study screening and selection.

SUPPLEMENTARY FIGURE S2

Risk of bias summary-ROBINS-I tool with traffic lights (A) and summary plot (B).
