# Supplementary figures and images for: Impact of periprocedural myocardial injury after transcatheter aortic valve implantation on long-term mortality: a meta-analysis of Kaplan-Meier derived individual patient data
Source: Front Cardiovasc Med. 2023 Nov 10;10:1228305. doi: 10.3389/fcvm.2023.1228305 (PMC10667910; doi:10.3389/fcvm.2023.1228305)

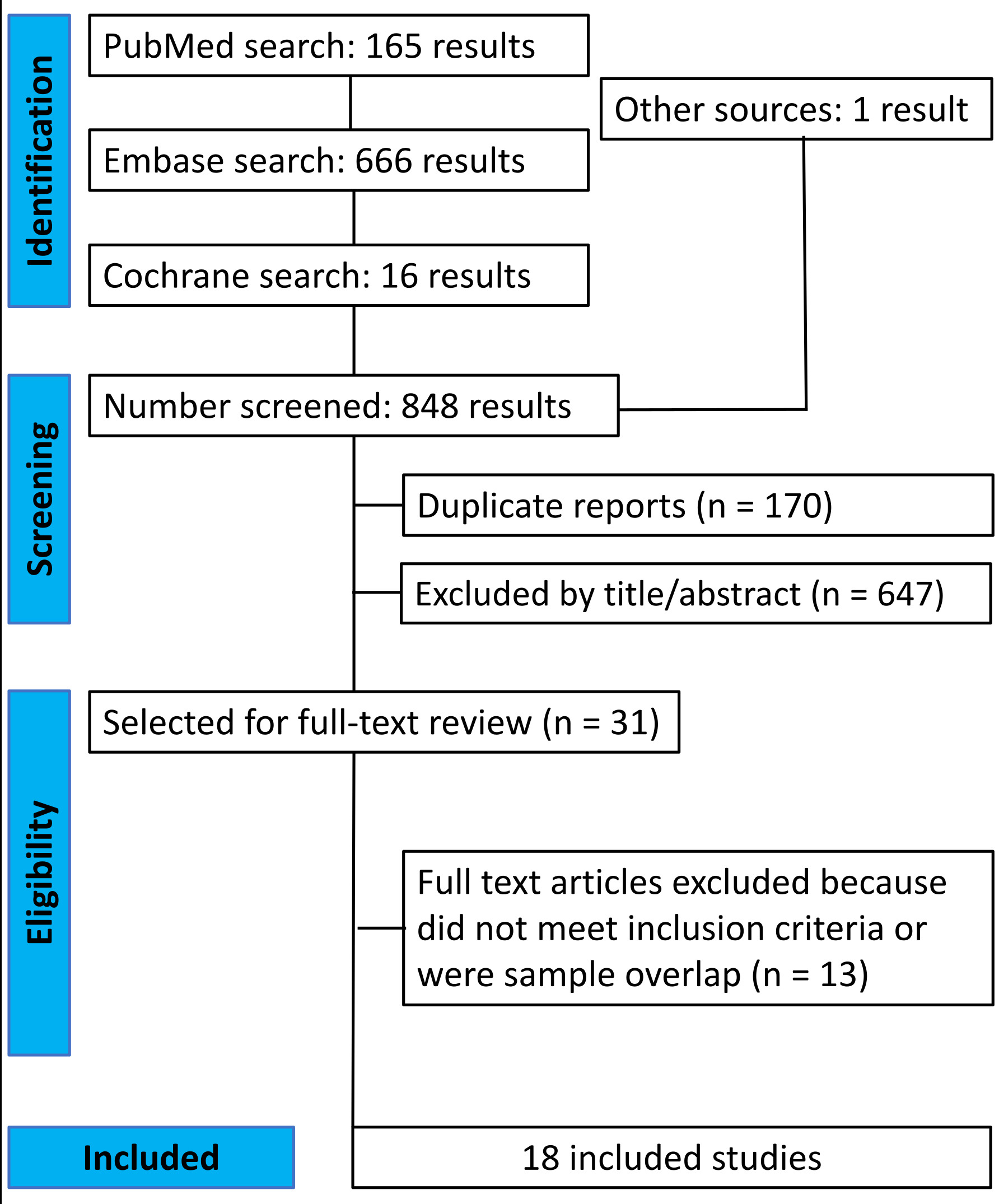

Supplement: Supplementary file 1 [file Image1.jpeg]

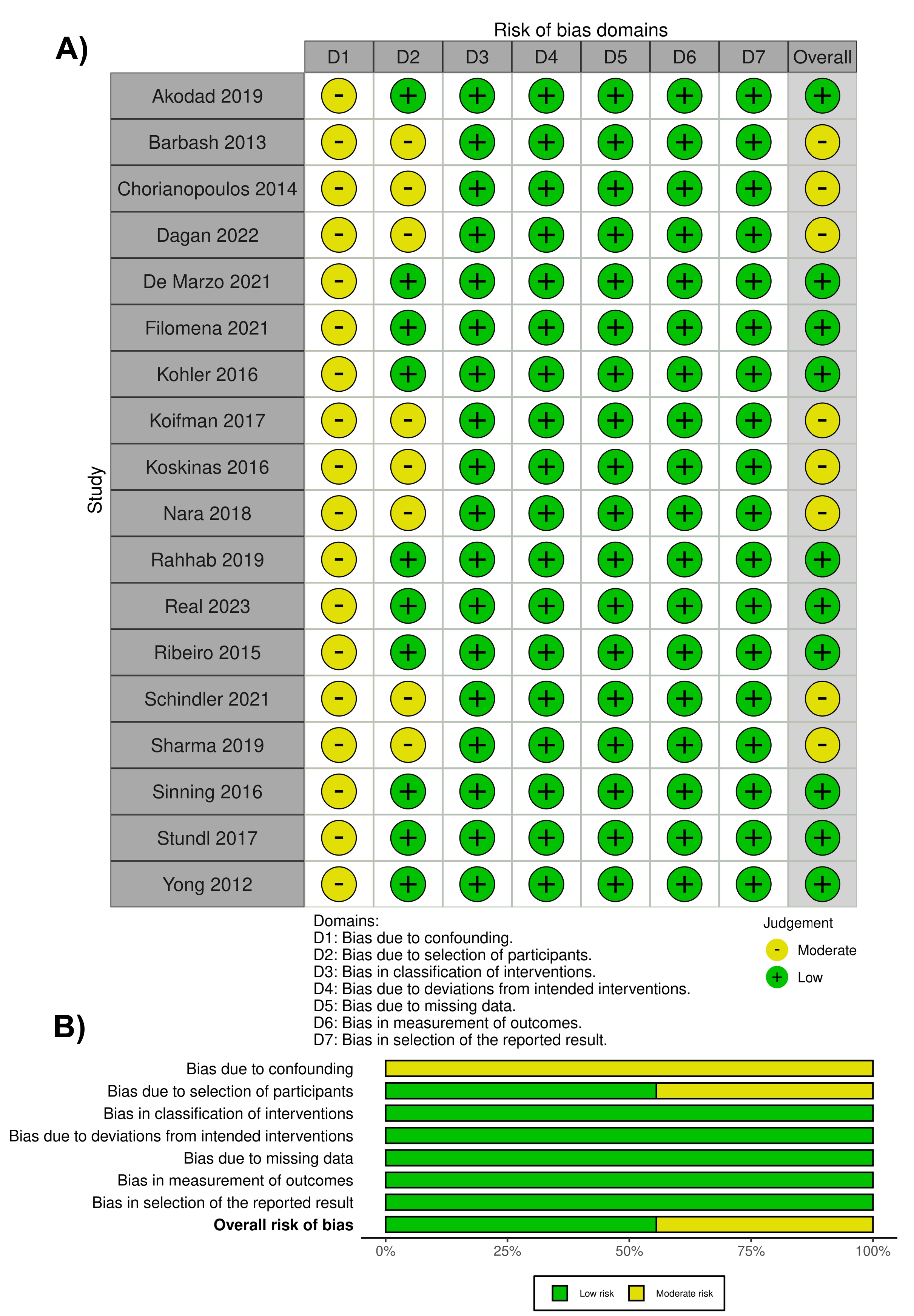

Supplement: Supplementary file 2 [file Image2.jpeg]
